# Developmental Abilities to Form Chunks in Immediate Memory and Its Non-Relationship to Span Development

**DOI:** 10.3389/fpsyg.2016.00201

**Published:** 2016-02-23

**Authors:** Fabien Mathy, Michael Fartoukh, Nicolas Gauvrit, Alessandro Guida

**Affiliations:** ^1^Bases Corpus Langage UMR 7320 CNRS, Université Nice Sophia-AntipolisNice, France; ^2^CHArt Lab, Ecole Pratique des Hautes EtudesParis, France; ^3^Centre de Recherches en Psychologie, Cognition et Communication, Université Rennes IIRennes, France

**Keywords:** chunking, short-term memory, immediate memory, working memory, cognitive development, capacity, algorithmic complexity, compression

## Abstract

Both adults and children –by the time they are 2–3 years old– have a general ability to recode information to increase memory efficiency. This paper aims to evaluate the ability of untrained children aged 6–10 years old to deploy such a recoding process in immediate memory. A large sample of 374 children were given a task of immediate serial report based on SIMON®, a classic memory game made of four colored buttons (red, green, yellow, blue) requiring players to reproduce a sequence of colors within which repetitions eventually occur. It was hypothesized that a primitive ability across all ages (since theoretically already available in toddlers) to detect redundancies allows the span to increase whenever information can be recoded on the fly. The chunkable condition prompted the formation of chunks based on the perceived structure of color repetition within to-be-recalled sequences of colors. Our result shows a similar linear improvement of memory span with age for both chunkable and non-chunkable conditions. The amount of information retained in immediate memory systematically increased for the groupable sequences across all age groups, independently of the average age-group span that was measured on sequences that contained fewer repetitions. This result shows that chunking gives young children an equal benefit as older children. We discuss the role of recoding in the expansion of capacity in immediate memory and the potential role of data compression in the formation of chunks in long-term memory.

A recoding process involves dividing an apparent arbitrary input into meaningful groups. The ability to recode rapidly inputs made of identical constituents (000.111), regular series (123.321) or known material (e.g., 25.12.1986, your favorite Christmas year) has long been studied to account for the recall of information over short intervals (Miller, 1956, 1958; Bower and Winzenz, 1969; Bor and Owen, 2007; Farrell, 2012; Mathy and Feldman, 2012; Mathy and Varré, 2013). Although this ability to chunk inputs has been thought to alleviate capacity limits in adults (Cowan, 2001; Cowan et al., 2004), it does not constitute a full explanation of capacity increases in immediate memory observed in children (Gilchrist et al., 2009), or age-related declines in immediate memory (Naveh-Benjamin et al., 2007; Gilchrist et al., 2008). It has been argued that capacity increases in memory during child development were due to a growth in the number of items that can be held independently of one another, not to an increase in chunk size (Gilchrist et al., 2009; Cowan et al., 2010a). The present study draws similar conclusions.

Although chunking seems related to the deliberate hierarchical reorganization of an input (Ericsson et al., 1980; Klahr et al., 1983; Servan-Schreiber and Anderson, 1990; Perruchet and Vinter, 1998; Gobet et al., 2001; Rabinovich et al., 2014; Solway et al., 2014), another origin is a more perceptual exploitation of statistical regularities in the environment (Perruchet and Pacteau, 1990; Servan-Schreiber and Anderson, 1990). Three types of regularities can be highlighted.

Firstly, co-occurrences of events can be found in the input stream. They can allow children to discover the words of a language (Saffran et al., 1996; Perruchet et al., 2014) without relying on particular explicit strategies used for instance by adults becoming experts (Chase and Simon, 1973a; Charness, 1979; Ericsson et al., 1980; Ericsson and Kintsch, 1995; Gobet et al., 2001; Hu and Ericsson, 2012). Tracking the transitional probabilities of an input such as “bidakupadotigolabubidaku” for instance allows a child to incidentally segment the input stream into words. These newly formed words can be used to chunk the previously encountered subsequences in a new input stream presenting the same statistical patterns.

A second possibility for forming a chunk is to take profit of a unitization process that does not depend on the co-occurrences of subsequences, but which rather depends on the task at hand (for instance, learning a particular sequence of motor actions *x*−*y*−*z* no matter its frequency; see Perlman et al., 2010; Minier et al., 2015). In this case, the *xyz* chunk allows the participant to speed up the motor actions, as when one retrieves the alphabet (Klahr et al., 1983).

A third way to form a chunk is to cluster together a set of identical units of a sequence sometimes presented just once, which has been referred to as a form of automatic or perceptual chunking (clustering can be used for instance to train pigeons to learn lists more rapidly, Terrace, 1987; see also Gobet et al., 2001). Some research suggest that infants are able to chunk object arrays when the objects are spatiotemporally grouped into two sets of two (Feigenson and Halberda, 2004) and toddlers are even able to quickly assign labels to such newly formed chunks (Kibbe and Feigenson, 2014). More diverse conditions were studied by Feigenson and Halberda (2008) to show that perceptual, conceptual, linguistic, or spatial cues are all sufficiently efficient to allow 14-month-old infants to store four-object arrays in memory. This literature suggests that an automatic process is available to children to recode information, which will serve as a working hypothesis in the present study to test if children have a general ability to recode information to increase memory efficiency regardless of age. To extend these previous studies, our goal was also to examine how information in working memory can be reorganized on the fly using a recoding process that can rely on external visual cues rather than internally stored knowledge.

Because conceptualizations of older children are more robust, we thought that reducing previously acquired knowledge would be best for studying how limited-capacity memory can be expanded by mentally organizing stimuli in working memory. The present study therefore investigates how working memory capacity is enhanced with the development of chunking abilities using a novel approach based on an old game (SIMON®). The original game consists of asking individuals to reproduce a sequence of colors that lights up by pressing four corresponding colored buttons (green, red, yellow, blue). The game starts with lighting one color at random and progressively increases the number of colors whenever the reproduction by the player is correct. Gendle and Ransom (2006) reported a procedure for measuring a working memory span using SIMON®, and they showed that the procedure is resistant to practice effects. Previous studies have shown that this setting has many advantages for measuring the memory span of different populations, including those with speech or hearing pathologies (Karpicke and Pisoni, 2004; Gendle and Ransom, 2006).

In our version of SIMON®, the chunks in the to-be-remembered sequences were not generated by an artificial grammar as in another study (Karpicke and Pisoni, 2004). Instead, we posed that the chunkability of the sequences could be measured by their algorithmic complexity, a notion that links the compressibility of a sequence to its simplicity. The algorithmic complexity of a sequence is the length of the shortest algorithm that is able to build the sequence (Kolmogorov, 1965; Li and Vitányi, 1997). In coding theory, the goal of an algorithm used for data compression is to attempt to find any kind of regularity in an input to encode, store and transmit information more efficiently without any loss of data (i.e., the uncompressed data is identical to the original data). From this perspective, a chunk can be seen as a new representation obtained with an optimally compressed code. Put simply, a sequence can be compressed when at least one chunk can be used to recode a regular subsequence into a smaller subsequence^1^.

When test circumstances allow a recoding process, the data compression approach can prove successful to account for how chunks can be formed rapidly without calling upon a consolidation process in long-term memory. Mathy and Feldman (2012) have for instance shown that compressibility in to-be-remembered sequences of digits was used by adult participants for immediate serial report. Their model estimated that the length of the compressed lists held in working memory was approximately 3–4 chunks, which corresponds to the working memory capacity limits found by many other investigators using diverse protocols hindering a chunking process (Cowan, 2001). However, their estimation of the capacity limit was also in line with the Miller's (1956) 7 ± 2 estimate because the 3–4 alleged compressed subsequences corresponded to seven uncompressed remembered items on average. Still, the more robust estimate across their experiments was the number of compressed lists (3–4), not the number of uncompressed items that instead varied freely depending on the manipulated compressibility of the sequences.

One basic idea is that a short algorithm such as “FOR *i* = 1:7, PRINT *xxy*” (or in somebody's mind, a more natural rule such as “7 × *xxy*”) can account for the low complexity of *xxyxxyxxyxxyxxyxxyxxy*. In comparison, the string *xyxxxyxxyxxxyxyyxxxxy* might require a trivial algorithm such as “PRINT *xyxxxyxxyxxxyxyyxxxxy*” (i.e., the string itself, when no regularity can be detected by the compression process). A minimum description length approach (e.g., Rissanen, 1978; Robinet et al., 2011) can be used to know whether the recoding of the subsequence *xxx* into a new symbol *z* can allow a greater compression of *xyxxxyxxyxxxyxyyxxxxy*. It could hardly be the case for the present example because “*z* = *xxx*, PRINT *xyzyxxyzyxyyzxy*” is globally not shorter than the original string (since the rewriting process includes “*z* = *xxx*”! But note that better recoding is not excluded). Although such a recoding process is not excluded in humans, it is not practical to estimate the compressibility of a short sequence.

One limitation of the minimum description length is that it deeply relies on the arbitrary choice of a particular coding language. A more neutral and objective approach particularly suitable for the short sequences used in the present study (i.e., containing less than a dozen symbols) is to approximate the algorithmic complexity by use of the coding theorem method (Gauvrit et al., 2014, 2015; Dieguez et al., 2015). This method is based on the fact that *K*(*s*) ≈ log_2_(*m*(*s*)), where *K*(*s*) is the algorithmic complexity of the sequence *s*, and *m*(*s*) is the probability that a randomly chosen deterministic algorithm produces *s*. The method uses the fact that the compressibility of a sequence is directly related to the probability that is randomly generated by an unspecified machine^2^. Unfortunately, this approach involves that we cannot know what the optimal compressed code is exactly.

To do so, we estimated the complexity of a set of random sequences of four colors using the *algorithmic complexity for short strings* (*accs*) R package (Gauvrit et al., 2014), and we split for each list length the simpler sequences from the more complex sequences to construct the chunkable vs. non-chunkable experimental conditions. The non-chunkable sequences will serve as a baseline for estimating participants' storage capacity (for instance, a capacity to retain four items would be four slots). The chunkable sequences will serve for estimating participants' storage capacity while chunking occurs (for instance, eight items). The ratio between the two estimates simply gives the number of items that can be packed into each slot on average (in the present case, 8/4 = 2 items per slot), or more simply put, this simply gives an estimate of chunk size.

Our working hypothesis was that if there is no chunking across age groups, one first basic prediction is that color items are recalled separately and that there is a simple growth of immediate memory capacity with age (Pascual-Leone, 1970; Burtis, 1982; Case et al., 1982). This is what we call the null scenario among the following set of predictions. A second case was that if an automatic chunking process is already available to infants and toddlers (Feigenson and Halberda, 2008) when a few redundant information is prompted into the task, the span of children across all ages for the chunkable sequences should increase in an additive manner. For instance, if older children are able to group perceptually two regular items into a new chunk, the same two items should be grouped by younger children, regardless of their span. For instance, for a *blue*−*blue*−*red*−*green* sequence, the youngest children would automatically encode and correctly recall *blue* − *blue* with a span of one chunk (let's hypothesize a similar correct recall of *blue* − *blue* no matter its position in the sequence). Older children would recall *blue* − *blue* − *red* with a span of two chunks, and the oldest would recall the four items with a span of three chunks. Another example is if the span increases from five to six in older children for the non-chunkable and the chunkable sequences, respectively, a group of younger children having a span of three for the non-chunkable sequences should show the same increase of one item for the chunkable sequences, which would allow them to reach a span of four on average. This prediction is based on the possibility that capturing a regularity does not involve any sort of computation or reorganization of the material to fit capacity. The two automatically grouped items into a chunk would not reflect an ability to reorganize information within each slot to multiply capacity. This case is additive in that there is a similar gain across age groups for the chunkable sequences. No interaction between age and the chunking factor should occur under this first additive scenario. However, this case operates only when a couple of items contain a regularity. If more regularities are present, there is a greater probability that the older children will encode more items, and as such, this would reflect the next following scenarios. For instance, a sequence *blue*−*blue*−*red*−*red*−*green*−*green* that contains too many regularities could not be encoded correctly with a span of one chunk but it could with a span of three.

A third case reflecting an absence of increase in chunking would show a similar gain in information reorganization at different ages. For instance, if older children are able to reorganize information to reach seven items for the chunkable sequences instead of five items for the non-chunkable sequences, they show a capacity to pack on average 7∕5 = 1.4 items per chunk. If the younger children can show the same ability based on a smaller starting capacity of three items for the non-chunkable sequences, they should show a capacity of 3 × 1.4 = 4.2 items for the chunkable sequences. This is a multiplicative scenario of capacity, but still this is an additive scenario on the log of capactity (i.e., because the multiplier effect is constant, the log of capacity shows an additive effect). For instance, *log*_2_(4.2) − *log*_2_(3) = 0.49, as well as *log*_2_(7) − *log*_2_(5) = 0.49. No interaction between age and the chunking factor should occur under this second additive scenario when taking the log of capacity.

Finally, if chunk size increases with age, the span for the chunkable sequences should increase more than multiplicatively with the span. This prediction would involve that a true reorganization process occurs within each of the available slots to form larger chunks with age. One possibility is that a group of younger children having a span of three for the non-chunkable sequences might show a span of three for the chunkable sequences on average, while older children may show a span of four for the non-chunkable sequences and a span of eight for the chunkable sequences. This simply means that the respective two groups (younger children, older children) would progressively increase the number of items packed into their available slots (respectively, one item per chunk and two items per chunk). Here, we consider the additive scenarios as the null hypothesis (no increase in chunk size) and the multiplicative scenario as the alternative hypothesis (increase in chunk size).

## 1. Methods

To study abilities to form chunks in immediate memory, our goal was to prompt a recoding process based on external cues rather than one based on internal knowledge (to avoid contribution from long-term memory). Children were administered a task of immediate serial report inspired of the SIMON®, a memory game made of four colored buttons (red, green, yellow, blue) requiring players to reproduce a sequence of colors within which repetitions could occur to induce a recoding process on the fly.

### 1.1. Participants

A total of 374 healthy children were split into five age groups: 6-year-olds (*M* = 6.1 years, *SD* = 0.20, *n* = 54), 7-year-olds (*M* = 7.0 years, *SD* = 0.26, *n* = 70), 8-year-olds (*M* = 8.0 years, *SD* = 0.28, *n* = 94), 9-year-olds (*M* = 8.9 years, *SD* = 0.29, *n* = 92), and 10-year-olds (*M* = 9.9 years, SD = 0.27, *n* = 64), from several public schools of the same county (note that most schools in France are public). Most children were from middle-class families. All of the children participated voluntarily, and their parents signed an informed consent form. None of these participants were colorblind.

### 1.2. Design

**Figure 1:**
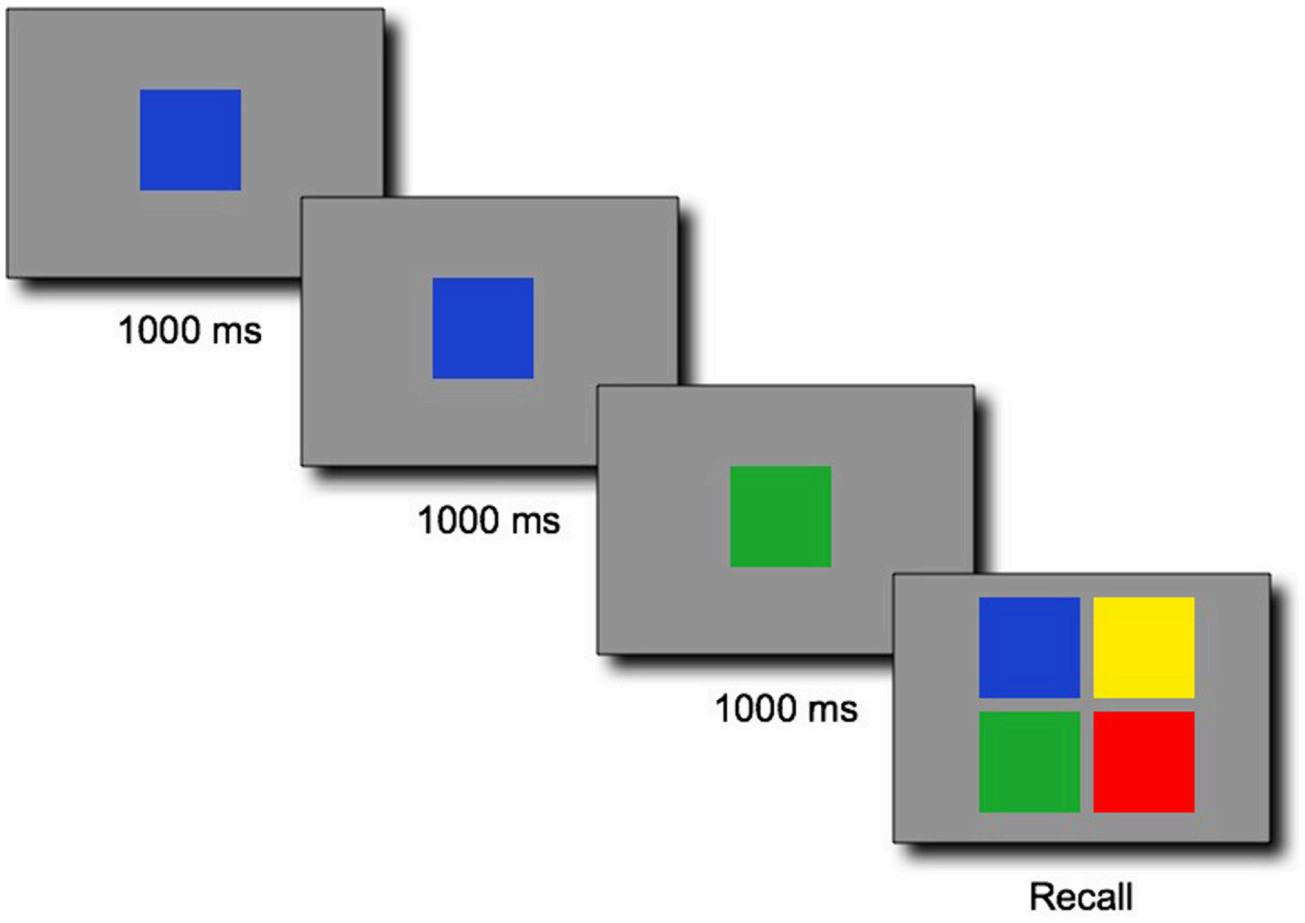
**Illustrative example of a sequence of three colors for the span task adapted from the SIMON® game, followed by a response screen in which the four colors were randomly placed on each of the four locations at each trial**. The 1000-ms timing was in reality split between a 400-ms presentation of the color followed by an inter-stimulus interval of 600 ms during which a blank gray screen was displayed.

The one to-be-remembered sequence consisted of a series of colored squares presented serially at the speed of 1000 ms per color (Figure 1). Each sequence started with a fixation cross centered on the screen for 1000 ms. Following the sequence, a recall screen displayed four colored buttons on which the participants were invited to click to recall the whole sequence in the correct order. Contrary to the original game, the task was nonspatial: each color item was presented in the center of the screen one after another. The main reason for running a nonspatial version of the task was that our method was not developed to measure the complexity of spatial patterns in 2D. Instead, it was developed to measure the regularity of a (1D) string of symbols. Spatial patterns such as “four colors clockwise,” “two diagonals,” “top row, bottom row” would also probably have interfered with the regularity in the sequences of colors that our metric captures. To further discourage spatial encoding, we randomly assigned the colors in the response screen to the four possible quadrants for each new trial. The colors were therefore never in the same locations in the response screens between trials. To mimic the original game during responses, the clicked colored pads were briefly lit up with lighter colors during 300 ms, as if a bulb was rapidly turned on. Clicking on the colors was paced by this lighting of the pad (the children could not click on the next color while the current pad was lit).

Feedback (“perfect” or “not exactly”) was displayed according to the accuracy of the response after the participant validated the response using the press bar. Feedback was given to tell the participant whether the response was correct or not. This insured for instance that the participants would remember that they were required to recall the series in the correct order.

The participants were given two complexity conditions (counterbalanced between participants). The Simple condition prompted a chunking process, while the Complex condition solicited less chunking opportunities. The sequences in each condition were taken from a random distribution of thousands of sequences for which we computed their algorithmic complexity. The sequences were then ranked according to their complexity, with the 100th percentile corresponding to the most complex sequences. The sequences in the Chunkable (i.e., simple) and Non-chunkable (i.e., complex) conditions were chosen at the 25th and the 75th percentile to constitute the two experimental conditions. Sequences around the lowest percentile would correspond to repeated sequences of the same color (e.g., “blue-blue-blue …”) and while 100th seemed a good choice to use the most complex sequences, we chose to restrict all of the sequences of length four and up to three colors only, which lowered their complexity. Our goal was to best match the number of chosen colors between the two conditions, and we thought that having four colors in the chunkable condition would not allow enough chunking opportunities to detect children's abilities.

The procedure matched a standard memory span task (e.g., the working memory subtests of the Wechsler Intelligence Scale for Children/Wechsler Adult Intelligence Scale) in which the length of the to-be-remembered sequences progressively increases, starting with length two. For each condition (Chunkable and Non-chunkable), there were two different sequences per length but the task automatically stopped when the participant was unable to correctly recall the two sequences of a given length.

Table 1 shows a sample of sequences given to participants. The two sequences of length two were identical in both conditions (e.g., *blue* − *red*, then *green* − *yellow*) in order to have the participants use all of the colors at least once during the first two trials, as a warm-up. Noteworthy is that these four colors are one-syllable words in French. Sequences of length three only contained two different colors in both conditions (e.g., *green* − *yellow* − *yellow* for a chunkable simple sequence, and *blue* − *yellow* − *blue* for a less-chunkable complex sequence). In both conditions, the sequences of length four and up never contained more than three different colors. One opposite pair (simple vs. complex) would be *yellow*−*blue*−*yellow*−*blue*−*blue* vs. *yellow* − *red* − *green* − *red* − *yellow*. The *yellow* − *red* − *green* − *red* − *yellow* is one example of a complex sequence that still contains a regularity since it is a palindrome. However, recoding the sequence as a palindrome probably requires more computation than simply grouping the contiguous identical colors in *yellow*−*blue*−*yellow*−*blue*−*blue* (not to mention that the palindrome is less noticeable when presented sequentially than when flattened out in Table 1). Note that we did not seek to avoid completely a recoding process of the complex sequences, since it was inevitable in some way for most sequences. Our compressibility metric only insured that the Simple condition was more compressible than the Complex condition on average.

**Table 1:** **Sample of sequences in the Simple and Complex conditions, up to seven items**.

**Length**	**Simple**	**Complex**
2 (warm-up)	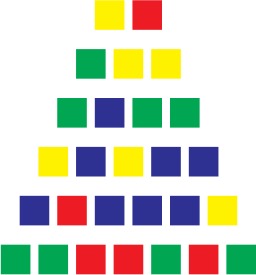	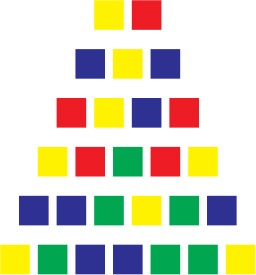
3		
4		
5		
6		
7		

*The Simple condition induces the formation of chunks, while the Complex condition corresponds to less chunkable sequences. The conditions were not limited to seven items but continued until reaching the stop criterion*.

### 1.3. Data scoring and data analysis

The span was calculated using the all-or-nothing method (Conway et al., 2005), in which credit was given to completely correct recalled sequences when both the items and the positions were correctly recalled (0.5 point per sequence correctly recalled; 1 point was automatically granted before starting the experiment for the virtual correct recall of one color). We also used the highest span method to measure the maximum level attained by the participant before failing. For instance, a participant was credited with a span of 1.5 when only one (out of two) sequence of two items was not correctly recalled, and when no other longer sequences were correctly recalled. The highest span method would however indicate a span of 2. To anticipate, we detail in the Results section why we eventually mainly focused on the highest span. To test the main hypothesis, these individuals' spans were submitted to a mixed analysis of variance (ANOVA) to test the interaction between the Chunkable vs. Non-chunkable factor and Age. We also computed the transitional error probabilities and the general effect of complexity on performance. The data is included in the Supplementary Material.

## 2. Results

First, we split the results according to task order (i.e., whether the task was the first one or the second one, regardless of the manipulated chunking factor). The respective spans for the entire sample of participants were respectively 3.8 (*sd* = 1.1) for the first task and 3.9 (*sd* = 1.2) for the second task. The respective spans using the highest span method were respectively 4.4 (*sd* = 1.2) for the first task and 4.4 (*sd* = 1.3) for the second task. We observed no difference between the first task and the second task, for either of the span measures [*ts*_(373)_ < 1.5, *p* > 0.14]. Measures of the span using the all-or-nothing method or the highest span method showed a high correlation (*r* = 0.93, *p* < 0.001, *N* = 374; including when partialling out Age, *r* = 0.92, *p* < 0.001, *df* = 371), and regardless of task order (the preceding statistics are given for the first task, but the statistical results were strictly identical for the second task). Given the high correlation between our two span measures, we chose to rely solely on the second measure (hereafter: Span) to conduct the following analyses.

**Figure 2:**
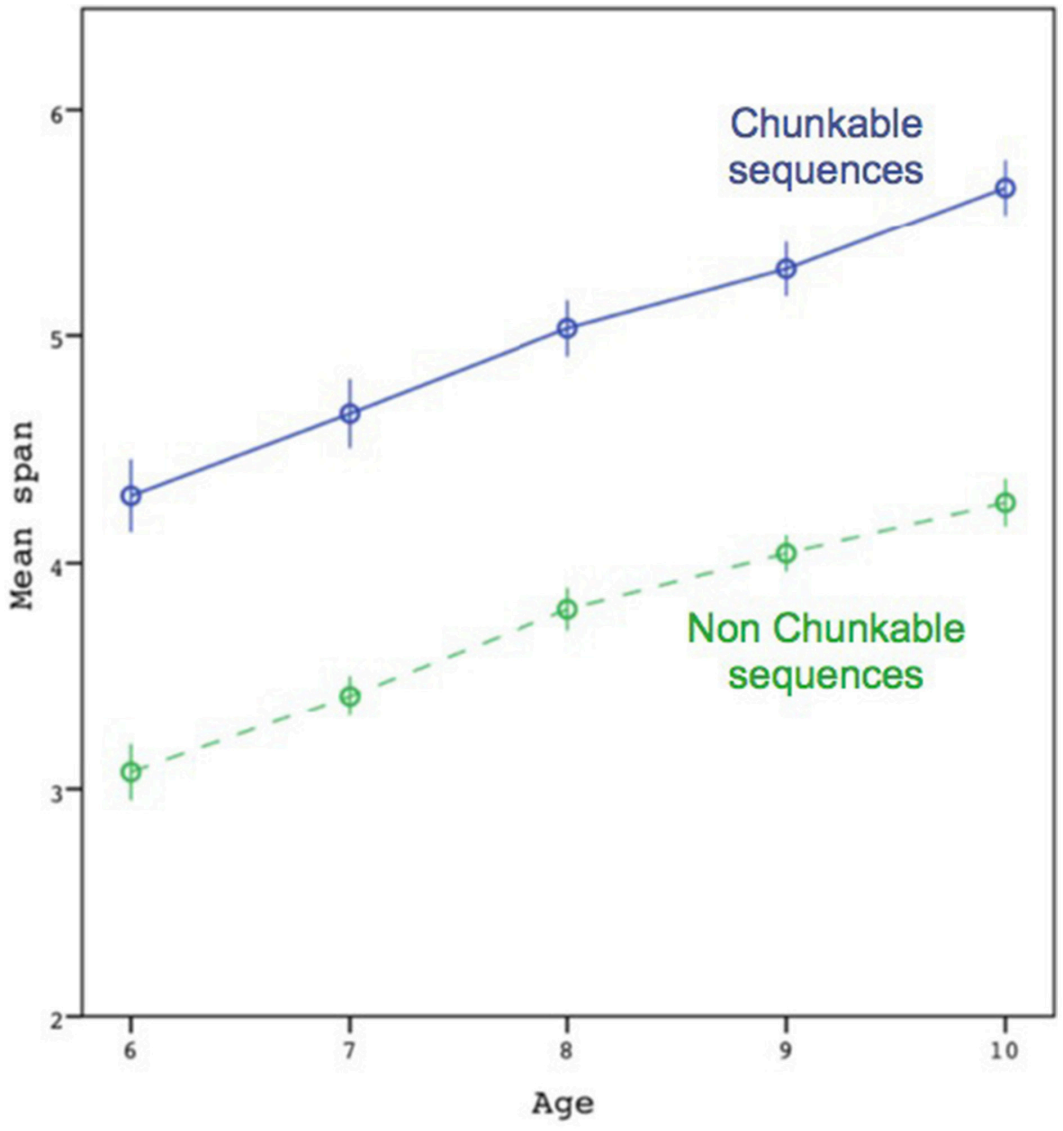
**Average maximal span attained by participants, as a function of age and chunkability of the sequences**. Error bars are ±1 standard errors. The dotted line represents the non-chunkable condition. The maximal spans on which the mean was computed were based on the individual's highest span.

Figure 2 shows the mean span as a function of age, separated by sequence type (chunkable vs. non-chunkable). The respective means across participants for the two conditions (non-chunkable vs. chunkable) of the Chunking factor were:

3.1 (0.9), 3.4 (0.8), 3.8 (0.9), 4.0 (0.8), 4.3 (0.8), vs. 4.3 (1.2), 4.7 (1.3), 5.0 (1.2), 5.3 (1.1), 5.7 (1.0).

The individuals' spans were submitted to a two-way analysis of variance (ANOVA) with Chunking (chunkable vs. non-chunkable) as a within-subject factor and Age (6, 7, 8, 9, and 10) as a between-subject factor. The results showed a clear effect of Age [$F_{\left(4\right)}=24,p<0.001,\eta_{p}^{2}=0.21$]; the Newman–Keuls test also showed systematic differences between contiguous ages, except between 8 and 9. Although the results also showed an effect of Chunking [$F_{\left(1\right)}=393,p<0.001,\eta_{p}^{2}=0.52$], we found no interaction effect between the two factors [*F*_(4)_ = 0.21, *p* = 0.93]. A significant interaction (including when taking the log of the individuals' spans) would have been interpreted as an increase in chunk size with age and would have confirmed the multiplicative scenario.

Although the gain in the chunkable condition in Figure 2 is significant according to the preceding ANOVA, it does not seem impressive (i.e., around 1.3 more colors recalled). Other evidence suggests that it is not the case. The gain of 1.3 colors seems more impressive in relation to the capacity limit found across age groups. For instance, there was no difference between the span attained by the 10-year-olds in the non-chunkable condition and the span attained by the 6-year-olds in the chunkable condition [*t*_(116)_ = 0.17, *p* = 0.87], mimicking a growth of 4 years in capacity. Moreover, in comparison to the average span of the 6-year-olds in the non-chunkable condition (3.1), 4.3 in the chunkable condition still represented a large increase of 39% (which was significant, as well as all other increases observed at 7, 8, 9, and 10 years-old; *ts* > 8.8, *ps* < 0.001).

Another way to approach the idea that chunk size did not increase with age was to divide for each subject their respective mean spans between the two conditions, before averaging across age groups. The average ratios showed that the number of items that could be packed in the chunkable condition (in comparison to the non-chunkable condition) was nearly constant around 1.4. The ANOVA computed on these ratios with Age as a between-subject variable was not significant [*F*_(4)_ = 0.74, *p* = 0.57], whereas the one-sample *t*-test showed that the average ratio was overall significantly greater than 1 [*t*_(373)_ = 17.9, *p* < 0.001].

We then focused on analyzing whether the repetition of colors facilitated recall with age. Again, we followed the hypothesis that the proportion of encoded repeated items should not increase with age if chunk size does not develop. The goal here was to study how the recoding process occurs incrementally [similar analyses based on transitional error probabilities have been run by Johnson (1969)]. The recoding process should allow children to optimize capacity by grouping similar items; that is, when compression is available. To calculate the proportion of contiguous pairs that was encoded (or not) within sequences, we only focused on the sequences that were not correctly recalled in serial order entirely (otherwise, the proportions would not vary whatsoever). Within each sequence, we calculated the number of items that were correctly encoded (or not) whenever the current item was similar to the previous one, and conversely, whenever the precedent item was different. The ordinal Gamma test for each of the crosstabs (Repetition vs. No-repetition, in Table 2) showed only a significant effect of Age on the increase of the proportion of encoded pairs when there was no repetition of the items within the pair. The highest and more constant proportions in the Repetition condition—which did not lead to a significant Gamma—tend to show that encoding repetitive items is a primitive process in children.

**Table 2:** **Proportion of items encoded conditional on the similarity to the previous item**.

**x**	**Repetition**			**No-repetition**		
**Age**	**Encoded**	**Missed**	**Prop**.	**Encoded**	**Missed**	**Prop**.
6	180	18	0.91	473	234	0.67
7	285	37	0.89	668	332	0.67
8	455	53	0.90	1033	458	0.69
9	465	37	0.93	1134	454	0.71
10	350	44	0.89	819	326	0.72

*This analysis is only based on the sequences that were not correctly recalled in the correct order*.

**Figure 3:**
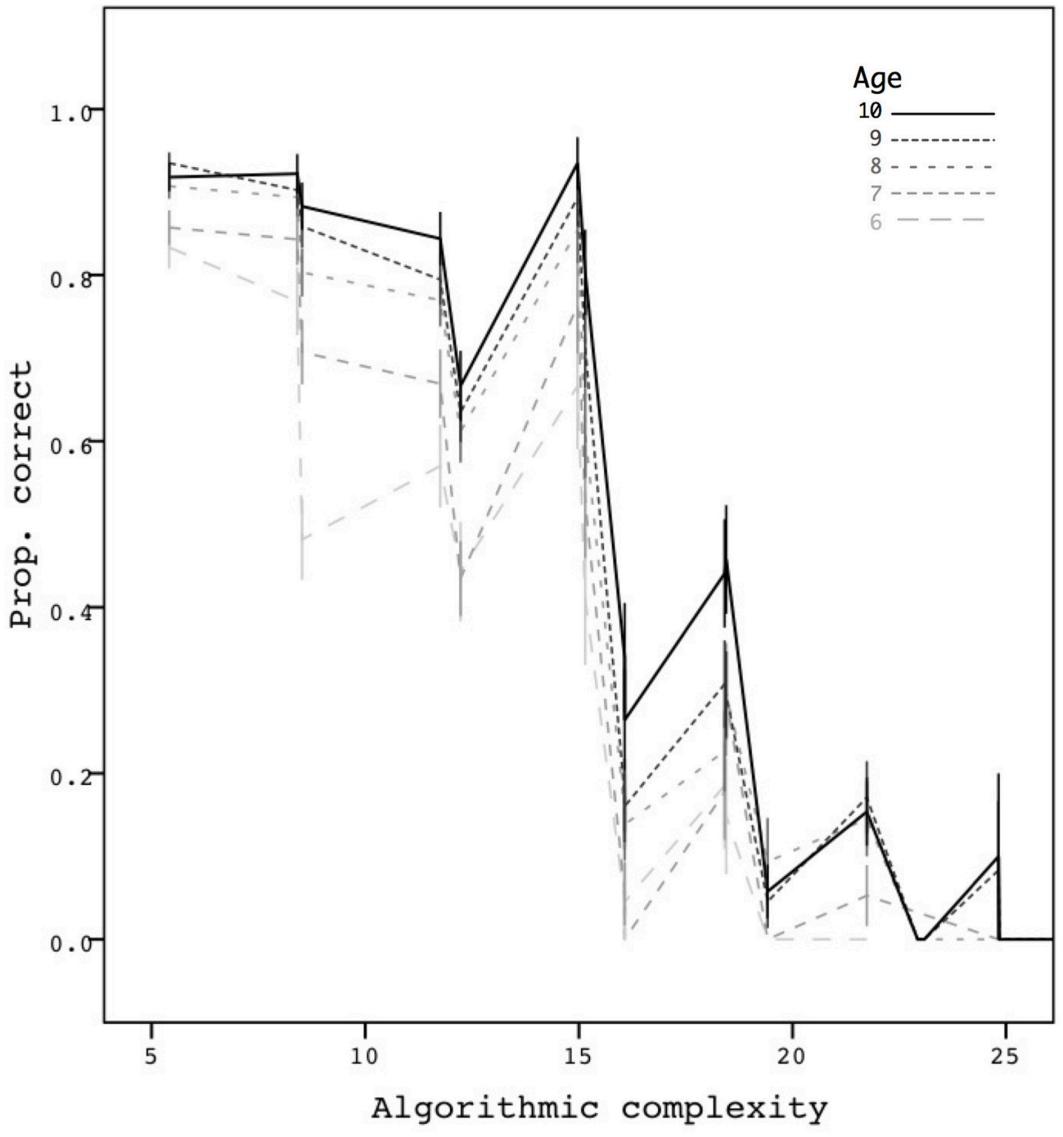
**Mean proportion correct as a function of complexity**. Error bars are ±1 standard errors. The mean proportion is based on 6580 trials.

Finally, Figure 3 shows performance as a function of the algorithmic complexity of the sequences and age, based on the 6580 trials. When we further restricted the data points to obtain a single average proportion for each age group and each complexity number, a simple correlation showed a clear decreasing trend as a function of complexity in a significant way (*r* = −0.86, *p* < 0.001, *N* = 83). Interestingly, the metric appears to take into account both the chunkability of the sequences and their length. Memorization can therefore be predicted in a continuous way by our compressibility metric.

## 3. Discussion

Although the maximum span length achieved at the electronic game SIMON® revolves around seven in adults (Gendle and Ransom, 2006), which recalls Miller's (1956) magical number, these seven items or so that adults can recall probably do not reflect seven separate chunks (see Cowan, 2005, chapter 3). Playing with the SIMON® looks like an ability to adapt to new situations in which one does not have much expertise, except that the ability to detect regular patterns might be primitive enough to allow rapid formation of chunks. Although how chunking takes place exactly in such a game remains difficult to approach, the primary goal of the current study was to observe age differences in recoding information in a task inspired by this game. We measured the compressibility of information in this task to study how information can be reorganized in working memory to fit capacity. If for instance only three colors can be remembered when no compression is allowed within the to-be-recalled sequence, it should recruit a capacity of three slots. If more colors can be recalled when information is more compressible (for instance, six), our idea is that the six colors still fit the three slots and that two colors on average are packed within each slot.

We evaluated the ability of children aged 6–10 years old to chunk on-line regular sequences of colors. To achieve this, we used a simple span task inspired of the SIMON® and a span was calculated for each child. One major modification was that unlike the real SIMON®, the location of the colors changed for each trial to avoid the spatialization of information. The chunkability of our sequences was measured by their algorithmic complexity, which was itself approximated by the coding theorem method (Gauvrit et al., 2014, 2015). The hypothesis was not that recoding is based on a Turing machine, but simply that the simplicity of a sequence that is estimated by the complexity of a deterministic machine can probably allow more chance for the participants to recode a sequence. Our estimation of the compressibility of the sequences of colors allowed us to build two experimental conditions. Our hypothesis was that the spans in the respective conditions would give us an estimation of the number of colors that can be chunked on average as a function of age. Two categories of sequences were used, simple ones (25th percentile of complexity) and complex ones (75th percentile of complexity). The former condition was made of easily chunkable sequences because colors were repeated in contiguous positions (e.g., *green* − *yellow* − *yellow*) or because other forms of regularities potentially rendered the sequences compressible (e.g., *yellow*−*blue*−*yellow*−*blue*), while the former condition was constituted by less chunkable sequences because fewer regularities could be extracted. Results showed a significant increase of performance as a function of age (6–10 years-old) and chunkability, but the key result was that no interaction was observed between the two factors. This result makes two important contributions that will be used as guiding lines to discuss our results. Firstly, even when complexity –and therefore the ability to recode information strategically– is controlled, the memory span still develops with age. Secondly, while the number of chunks increases as a function of age, the size of chunks remains stable. Finally, when chunking occurs, it generally concerns about two items only (since we hardly observed a gain of a couple of items for the chunkable sequences overall). We observed no strong interaction between age and the chunking factor which could have for instance shown an increasing chunk size with age (even after taking the log of the individuals' spans to reject an interaction due to a constant chunk size across age).

### 3.1. Immediate memory capacity development

If one wants to comprehend the growth of immediate memory capacities across development, it is important to know exactly what one is measuring through immediate memory tasks. As emphasized by Cowan et al. (2015), it is well-known that when one tries to measure immediate memory capacities, various factors can impact performance. Hence, a great deal of research these last 40 years has been to identify the key factors that determine immediate memory capacity. An important effort has been put into controlling some of these factors to verify whether immediate memory capacity still grows with age when these factors are neutralized, in particular the knowledge factor. Effectively, chunking in general refers to the amount of information that can be stored by meaningfully grouping information. Case et al. (1982) famously showed that if one controls the familiarity of the items (which operationalizes knowledge), no developmental differences in immediate memory appear in a word span when comparing adults to children (here 6 year-olds). However, since then, results have more often shown that immediate memory development is not entirely due to knowledge, and Case himself proposed another interpretation of his own results (for this rebuttal see Case, 1995). The same year, Burtis (1982) observed that when chunking strategies were controlled using matrices of letters, there was still a growth of immediate memory capacity with age. Cowan and colleagues in several other studies (Cowan et al., 2010b, 2011, 2015) have systematically controlled various elements that could explain the growth of immediate memory capacities. Cowan et al. (2010b) showed that even when one controls the ability to exclude less-relevant items, age differences in terms of immediate memory remain. Similarly when encoding differences are neutralized, immediate memory differences still exist between age groups (Cowan et al., 2011). More recently, Cowan et al. (2015) controlled familiarity, which enabled to manipulate the size of chunks through the use of knowledge. They used visual arrays of English letters vs. unfamiliar characters with a probe item recognition paradigm. Even with unfamiliar characters, that is, when the contribution of knowledge was minimal and therefore when chunking was minimal, there still was an increase of immediate memory capacities from first grade (7 years-old) to seventh grade (12 years-old). This increase was comparable to the one observed with English letters. As resumptively put by Cowan et al. (2015), whatever type of control is adopted, immediate memory seems to always increase with age.

Our study controlled the strategic factor by reducing the possibility to form chunks (in the condition for which compressibility was minimal). In contrast to previous studies, we believe that our task better reduces the role of acquired knowledge. Our results are identical to those of Cowan and colleagues: when we reduced potential chunking strategies, we still observe an increase in span with age. However, it must be noted that our manipulation draws on a different kind of chunking. According to Gobet et al. (2001), chunking can be dichotomized into two categories depending on when chunking occurs. The first category assumes a goal-oriented chunking (e.g., Miller, 1956), thought to be deliberate, where conscious strategies can play an important role. The second is perceptual chunking (e.g., Chase and Simon, 1973a), which is putatively more automatic and implicit, and which can be primitive (Feigenson and Halberda, 2008). As presented in the introduction, we believe that the repetition of colors enables the participants to heavily rely on such perceptual chunking by exploiting the statistical regularities of the repeated colors, but strategic factors can also be developed consciously. In the case of letters, the recoding possibilities seem more goal-oriented. Even if neither of the two types of chunking –in our works or in Cowan et al.'s (2015)– are entirely perceptual or goal-oriented, we believe that our task draws more on perceptual chunking than Cowan et al.'s (2015). Hence for the first time, our results show that even when one controls for perceptual chunking (which is reduced in our complex condition, thus allowing minimal rapid re-encoding of information on the fly), there still is an increase of immediate memory capacity with age. Our findings adds one more controlled factor to account for the growth of immediate memory capacity, after knowledge differences, encoding differences, and resistance to interference differences. One conclusion is that our observation that there is a progressive increase of capacity with age in our non-chunkable condition makes clear that the capacity to rapidly form chunks on-line does not solely account for the growth of immediate memory capacity. Something else is at stake to support the growth of the memory span. Conversely, our observation that there is not a greater progressive increase of capacity with age in the chunkable condition –than in the chunkable condition– means that chunk size cannot easily be increased by direct manipulation of information in immediate memory by children less than 10 years old. Chunks formed in long-term memory are probably a unique result of a consolidation process and children are probably better off using knowledge (i.e., chunks previously formed) to increase capacity than trying to re-organize the new incoming information on the fly.

### 3.2. Number of chunks increases with development but not size

Our originality was also to use a similar material (i.e., simple sequences of colors) for the chunkable vs non-chunkable conditions (whereas Cowan et al., 2015, for instance compared the span for chunkable letters against non-chunkable unfamiliar characters). The main result of the current study is that we found no increase of chunk size with age, a result which could have been observed had we obtained a significant strong interaction (i.e., on the log of the spans) between the Chunking factor and Age. The hypothesis was that repeated colors could be bound into chunks more easily by older children if their ability to optimize memory resource was based on a growing ability to compress information within chunks. The alternative hypothesis was that an automatic process is available to children to recode information when they can rely on external visual cues rather than internally stored knowledge. We found that the amount of information retained for the groupable sequences did not depend multiplicatively on the average age-group span that was measured on sequences that contained fewer repetitions. The gain for the chunkable condition was small and constant across age groups.

Across adulthood, it is well-known that the number of chunks in immediate memory does not vary, no matter practice (Tulving and Patkau, 1962; Cowan et al., 2004; Chen and Cowan, 2005; for a review, see Cowan, 2005) or expertise (e.g., Gobet, 1998; Lane et al., 2001; or to a very limited extent, Gobet and Simon, 2000). Instead, what does vary, is the size of chunks: they become larger with practice (Cowan et al., 2004; Chen and Cowan, 2005) and expertise (e.g., Chase and Simon, 1973b; Gobet and Simon, 1996a,b; Guida et al., 2012). For children, the story is different and more complicated. Some studies, like ours, shows that with age, the number of chunks increases while their size remain stable. For example, Gilchrist et al. (2009) investigated the development of working memory and chunks by manipulating the number, the size and the meaning of sentences. The size of the chunks was operationalized by the proportion of each sentence recalled, and the number of sentences recalled, enabling the authors to tap the number of adopted chunks. The authors observed that the chunk size was stable from the first group (7-years-olds) to the last one (young adults), while the number of chunks or slots in immediate memory increased. Interestingly, even if our operationalization of the size and number of chunks is at odds with that of Gilchrist et al. (2009), we found exactly the same results. The average ratio we calculated showed that the number of items that could be packed in the chunkable condition in comparison to the non-chunkable condition was stable across the different groups. The knowledge that children obviously accumulate in long-term memory does not seem to transfer to re-encoding information on the fly to form new associations, which stays stable in our study, and might be primitive. Using the repeated colors, chunk size could not depend on the participant's knowledge much. Instead, we show that encoding regular sequences is a primitive process in children (see for instance our analysis on how the pairs of colors were encoded equally well when the items were repeated, regardless of age), and we believe this process can play an important role in the formation of chunks in the long-term.

Even if our results conceptually replicate those of Gilchrist et al. (2009), opposite results do exist. Ottem et al. (2007) for example found that immediate memory span development reflects an increase in the size of each chunk rather than an increase of the number of chunks. This conclusion was drawn from three studies with participants' age ranging from 3 to 16 years-old. When the correlation between immediate memory span and language abilities was taken into consideration, no evidence of a relation between immediate memory span and age was observed. Interestingly, this is also what Jones and colleagues (Jones et al., 2008, 2014) found using CLASSIC and EPAM-VOC. These two computational models based on chunking were used to simulate the acquisition of vocabulary. In both cases the capacity of the models to simulate the increase of non-word repetition was independent of working memory size. The crucial factor to account for the data was long-term memory knowledge, and this showed that the size of working memory increases thanks to the increase of chunks size, not the number of chunks. Also relating to language abilities, Hulme et al. (1984) showed a linear relationship between speech rate and memory span in both children and adults, supporting the idea that increase of speech rate with age could account solely for the development of the memory span (i.e., a greater number of sounds can be contained in a two second period with faster articulation). This account is not incompatible with a growth of memory capacity. This results could effectively be reinterpreted as the idea that greater speech rate can increase what can be encoded within chunks. Regarding our study, if so, a greater speech ability with age should have facilitated the articulation of several repeated colors instead of switching colors. Such a process would have favored the chunkable sequences, but such an effect was not observed.

### 3.3. Limitation and the way forward

One limitation of the present study is that the modeling of compressibility is unable so far to determine the exact number of adopted chunks with age. Although the compressibility metric is useful to apprehend the compressibility of a whole sequence, it does not compute the number of actual chunks within a sequence. Computing the exact number of separate chunks that allow an optimal recoding of a short sequence is a line of research for the future. In the present study, analysis of the response times (to detect chunk boundaries) would be untestable since the repetition of colors necessarily involved quicker clicks of the mouse. One further line of experiments would be for instance to systematically vary the location of the colored pads after each click, to better understand the actual internal processes involved in forming chunks (see Gilchrist, 2015, for other methods such as adding neurophysiological measures to chunking research, which could be combined with models of compressibility to determine better the processes involved in the formation of chunks). Such experiments would be a more sophisticated way to predict how many items can be combined to form new chunks on-line.

## Author contributions

FM designed the research; MF performed the research; FM analyzed data; FM, MF, and AG wrote the paper. NG computed the algorithmic complexity.

### Conflict of interest statement

The authors declare that the research was conducted in the absence of any commercial or financial relationships that could be construed as a potential conflict of interest.

## Footnotes

^1^One limitation of this approach is that it was not developed to determine the exact chunks in a string. An optimal compressed code does not necessarily involve chunks. For instance, if a sequence *xyxyyy* is found more compressible than another one of the same length, the algorithmic complexity approach cannot determine whether the string should be recoded based on *xy*−*xy* − *yy*, *x* − *y* − *x* − *y* − *y* − *y*, or *xyx* − *yyy*, etc. The extraction of the optimal chunks in a string is a further line of research that we are considering in our lab. Below, we explain why we can only obtain an estimation of algorithmic complexity that is not based on an actual recoding process.

^2^Note that (1) algorithmic complexity is a measure of *random complexity*, not of *structured complexity*. This means that a string has high algorithmic complexity when it is random or shows no particular regularity or structure. Thus, algorithmic complexity shall not be confused with other measures, such as Bennett's logical depth, which is intended to capture structural complexity. For instance, the decimals of π can be produced by short algorithms such as the recursive Leibniz formula, and thus have a low algorithmic complexity. Yet, the algorithm computing the π decimals needs a long time to produce new decimals: so, the series has a large Bennett's logical depth. A direct comparison of algorithmic complexity and Bennett's logical depth in short strings revealed a lack of correlation when controlling for the length of the string (Soler-Toscano et al., 2013). (2) Contrary to Bennett's logical depth, algorithmic complexity is a direct measure of how compressible a string is, and is therefore particularly relevant to our present study. **(3)** Unlike measures based on specific compression algorithms such as the Lempel-Ziv method, algorithmic complexity makes no assumption about what type of compression method is required, but nevertheless determines how much an optimal algorithmic would reduce the string.
